# Supplementary material for: A multiresolution mixture generative adversarial network for video super-resolution
Source: PLoS One. 2020 Jul 10;15(7):e0235352. doi: 10.1371/journal.pone.0235352 (PMC7351143; doi:10.1371/journal.pone.0235352)
Supplement: S2 Data — (ZIP) [file pone.0235352.s002.zip › Editage Editing Service/Letter_from_the_Editor.docx]

尊敬的作者：

感谢您选择与Editage合作！

请您在已润色的文件中查看修改和批注。

如果您对润色内容有疑问，或收到期刊审稿人审稿意见，或者希望我们检查您所做的其他修改，请使用您EditageOnline^TM^帐户上的后续支持选项（<http://app.editage.cn/>）。

我们想了解您对我们的服务有什么看法，以及我们如何才能做得更好。请通过您的EditageOnline^TM^帐户将您对服务的反馈发送给我们。

**致谢编辑支持**

不少作者在他们文稿中表达了对Editage编辑支持的感谢。根据著名的国际医学期刊编辑委员会（ICMJE）发表指南中关于作者署名、润色或写作支持， 发表文稿中应给与致谢。这样的致谢也有助于向期刊编辑/审稿人保证，英语已经被彻底审查过，并且符合发表所需的标准。

如果您想致谢我们对这篇论文的编辑支持，您可以在论文的“致谢”部分加上以下句子：*We would like to thank Editage (www.editage.cn) for English language editing.*

致敬

您的编辑

| **Additional notes** |
| --- |

**Formatting**

(To ensure that your paper conforms to the formatting requirements of the target publication)

I have used the following webpage, along with the latest articles published by PLOS ONE, to make formatting adjustments: <https://journals.plos.org/plosone/s/submission-guidelines>. Because you have not attached author information in your Word document, this will need to be added to the document before submission. Please refer to my comments and the webpage above for further guidance on how to layout this information.

1. Title page: I have attached a template so that you can copy the desired format for the title page with information that was not provided in this study ( the author information).
2. Author information: After the title, a list of the authors of this study must be written. Please refer to previous PLOS ONE articles and this webpage (<https://journals.plos.org/plosone/s/submission-guidelines#loc-author-list>) on how to layout your names. Please also select a corresponding author (an author from the study who will be the main point of contact for the journal).
3. Figures: Figures (excluding any Tables) must not be included in the main manuscript file, but should be submitted as individual files. The individual figure file requirements are detailed here: <https://journals.plos.org/plosone/s/figures#loc-figure-file-requirements>. I will leave the figures in the document so that it is easier for you to understand any figure-related changes I have made, but you must delete them from the file before submitting your article to the journal. Figure captions should not be part of the individual figure files, but should remain in the main manuscript file. If I have repositioned any figures, this is so that the figure captions appear after the first paragraph the figure is mentioned in.
4. References: Please ensure that all your references use the “Vancouver” style and are referenced uniformly.
5. Section headings: I have changed the sizes of your headings and subheadings in accordance with the guidelines set by the journal, so that headings and subheadings can be easily distinguished.
6. Striking Image: You can choose a figure from your article or supporting information to be designated as the striking image (the image which is placed beside your article when searched or advertised on the journal webpage). If you do not submit one, then journal editors will choose one for you. Information on an appropriate striking image can be found here: <https://journals.plos.org/plosone/s/submission-guidelines#loc-striking-image>
7. Supporting information: Submissions are only accepted by this journal in English, but if this article was originally written in another language before this current English version, you can submit the original article as supporting information. This could help ensure that your intended meaning is clear. Such a document is only supplementary and is not necessary for your article to be published. You can consult the webpage below if you believe such a document would be beneficial (<https://journals.plos.org/plosone/s/supporting-information>).

**Figures and tables**

(Recommended changes to embedded figures/tables, which I was unable to modify)

1. Figures 1-4: I would recommend editing Prelu to ‘PReLU’, so that you use the standard abbreviation.
2. Figures 5-10: It is important to include axis titles, to aid a reader’s understanding of a graph. Example axis titles could be ‘Data sets’ on the x-axis, and ‘LPIPS evaluation results’ on the y-axis (or ‘tLP evaluation results’). Units should be included in brackets beside the axis titles, unless the values are dimensionless, in which case, the fact they are dimensionless should be stated somewhere in the main body of text.
3. Figure 11: There should be a space between a word and a bracket, e.g. ‘Bicubic (0.55)’. Moreover, I would recommend removing ‘(LPIPS)’ from your figure, with a simple line as part of your figure caption sufficing.

| **Quick tip** |
| --- |
| **Guideline** |
| Once abbreviations are introduced, it is important to use the abbreviation in the rest of your study. |
| **Explanation** |
| Readers may become confused if you fluctuate between using and not using the abbreviation. It is important to remain consistent throughout your work. |
| **Example** |
| For example, you abbreviate ‘video super-resolution (VSR)’ in such a way, and then proceed to use the full term ‘video super-resolution’ unnecessarily later in your work. Writing ‘VSR’ for the remainder of your main body of text is suitable. |

Thank you for choosing Editage for your manuscript editing needs; I enjoyed learning about your research whilst editing your study! I have tried to give as much detail in my comments, in the Word document and in this LFTE, to describe my reasons for editing certain parts of your work, as well as providing some ideas for some more substantial editing that you could consider. I would be grateful if you could provide any positive or constructive feedback you may have about my editing; it would be much appreciated.
