# Supplementary material for: A multiresolution mixture generative adversarial network for video super-resolution
Source: PLoS One. 2020 Jul 10;15(7):e0235352. doi: 10.1371/journal.pone.0235352 (PMC7351143; doi:10.1371/journal.pone.0235352)
Supplement: S2 Data — (ZIP) [file pone.0235352.s002.zip › Editage Editing Service/PLOS_ONE_Editage.docx]

RESEARCH ARTICLE

**A multiresolution mixture generative adversarial network for video super-resolution**

# Abstract

Generative adversarial networks (GANs) have been used to obtain super-resolution (SR) videos that have improved visual perception quality and more coherent details. However, the latest methods perform poorly in areas with dense textures. To better recover the areas with dense textures in video frames and improve the visual perception quality and coherence in videos, this paper proposes a multiresolution mixture generative adversarial network for video super-resolution (MRMVSR). We propose a multiresolution mixture network (MRMNet) as the generative network that can simultaneously generate multiresolution feature maps. In MRMNet, the high-resolution (HR) feature maps can continuously extract information from low-resolution feature maps to supplement information. In addition, we propose a residual fluctuation loss function for video super-resolution. The residual fluctuation loss function is used to reduce the overall residual fluctuation on SR and HR video frames, to avoid a scenario where local differences are too large. Experimental results on the public benchmark data set show that our method outperforms the state-of-the-art methods for the majority of the test sets.

# Introduction

Super-resolution imaging (SR) techniques are used to solve the classic problem of recovering high-resolution (HR) images from low-resolution (LR) images. These techniques are widely used in image processing. At present, there are many ways to obtain SR, but there is still room for further development to improve upon the techniques.

With the relatively recent development of artificial intelligence, the use of deep learning to achieve SR has attracted widespread attention [[1-8](#_ENREF_1)]. Many deep learning-based image methods are superior to traditional methods, achieving breakthroughs in the peak signal-to-noise ratio (PSNR) and structural similarity index (SSIM) metrics [[9](#_ENREF_9)]. Among them, image super-resolution (ISR) based on generative adversarial nets (GANs) [[10](#_ENREF_10)] has recorded improvements in visual perception quality. However, using adversarial training for video super-resolution (VSR) has not received the same attention. Unlike ISR, VSR has to consider the relation between consecutive video frames, considering both spatial and temporal information to generate results with temporal consistency and spatial consistency. The creators of VSR algorithm TecoGAN [[11](#_ENREF_11)] proposed a spatio-temporal discriminator and a Ping-Pong loss function to achieve such consistency. Using GANs in VSR can generate coherent and clear video details, however, there is still a large difference between the SR video implemented by TecoGAN and the real video. The performance of TecoGAN still needs to be improved, especially in areas with dense textures.

A multiresolution mixture generative adversarial network for video super-resolution (MRMVSR) is proposed in this paper. In order to make full use of the information of LR video frames and generate results with better visual quality, this paper proposes a multiresolution mixture network (MRMNet) for VSR. Contrary to the traditional network, the MRMNet has simultaneous multiple resolution feature maps during the training process, which can continuously extract information from the LR feature maps to supplement the HR feature maps. The LR frame is gradually enlarged to the target resolution after being put into the network. Moreover, we propose a residual fluctuation loss function, to avoid excessive local differences in the generated frames and to generate better results in areas with dense textures. The experimental results on public datasets show that the proposed method can effectively improve the performance of VSR, outperforming other state-of-the-art methods.

In summary, the contributions of the proposed method are summarized as follows: 1) an MRMNet is proposed for VSR, which makes full use of the information from LR images, 2) a residual fluctuation loss function is proposed for VSR to improve the visual perception quality of the resulting image, and 3) the performance of the MRMVSR model is fully evaluated, is superior to the performance of state-of-the-art video super-resolution methods.

There are five sections in this paper. We have briefly introduced the research content in Section 1. Section 2 examines related studies which motivate the proposed method. Section 3 introduces the proposed method in detail. In Section 4, the results and corresponding discussion of the experiments are presented, with concluding statements given in Section 5.

# Related work

In recent years, image and video acquisition, processing, and analysis have commanded greater focus from researchers [[12-16](#_ENREF_12)]. A significant amount of work has taken place analyzing ISR, with Wang et al. [[16](#_ENREF_16)] producing a comprehensive review of this body of work. The SR convolutional neural network [[15](#_ENREF_15)] proposed by Dong et al. uses deep learning for the first time in the field of ISR, accomplishing single image super-resolution, surpassing the traditional methods in terms of the PSNR and SSIM metrics. Researchers have looked to improve image quality by predominantly making improvements to the network, continuously accelerating the training process and hence improving the performance of ISR. The network structure of improved models for VSR can be divided into three distinct types: network front-end upsampling, network back-end upsampling, and iterative upsampling [[4](#_ENREF_4), [8](#_ENREF_8), [17-20](#_ENREF_17)]. Although these methods have achieved excellent results according to PSNR and SSIM, the visual perception quality is still poor. Several models follow SR GANs [[19](#_ENREF_19)] to combine the generative adversarial network and perceptual loss [[21](#_ENREF_21)] to obtain ISR, which can generate improved results in visual quality [[22-25](#_ENREF_22)].

VSR differs from ISR by requiring the generation of continuous multi-frame images, with a certain relationship between frames. Simply using ISR methods for VSR will lose related information between adjacent frames. VSR has been realized by complex calculation methods that consequently have very high computational complexity [[26](#_ENREF_26), [27](#_ENREF_27)]. However, most existing methods that use a deep-learning technique to complete VSR divide the task into multiple sub-tasks. Each sub-task recovers an HR image from multiple LR video frames [[1](#_ENREF_1), [3](#_ENREF_3), [7](#_ENREF_7), [28](#_ENREF_28)]. One such method, frame-recurrent VSR (FRVSR) [[29](#_ENREF_29)], takes multiple frames of LR images as input, learning motion compensation information through a stream evaluation network. This method uses the generated HR video frames to cyclically generate subsequent video frames, that can reuse high-frequency details and improve temporal consistency. Generative adversarial networks for ISR have been successful in improving visual perception quality; however few studies apply such a method for VSR. To address this problem, TempoGAN [[30](#_ENREF_30)] uses a generative adversarial network for VSR, improving overall temporal consistency. Furthermore, the TecoGAN algorithm introduced a novel spatio-temporal discriminator that gets rid of the single function of the authenticity identification, and can guide the network to generate spatio-temporal consistent results. Experimental results have found that TecoGAN can generate clear and coherent details.

Although existing methods produce improved video clarity, the performance of the existing methods in dense texture areas is still not satisfactory. Moreover, the visual perception quality needs to be further improved. In order to improve the performance in texture-dense regions and generate results with better visual perception quality, this paper proposes an MRMNet and a residual fluctuation loss function.

# Methodology

In this section, we introduce our proposed method that consists of network architecture, MRMNet, and the residual fluctuation loss function that will be used for training purposes.

**MRMNet**

To make full use of the information from LR frames such that the quality of VSR improves, we propose an MRMNet. This network architecture, a generator network in adversarial training, is illustrated in Fig 1.


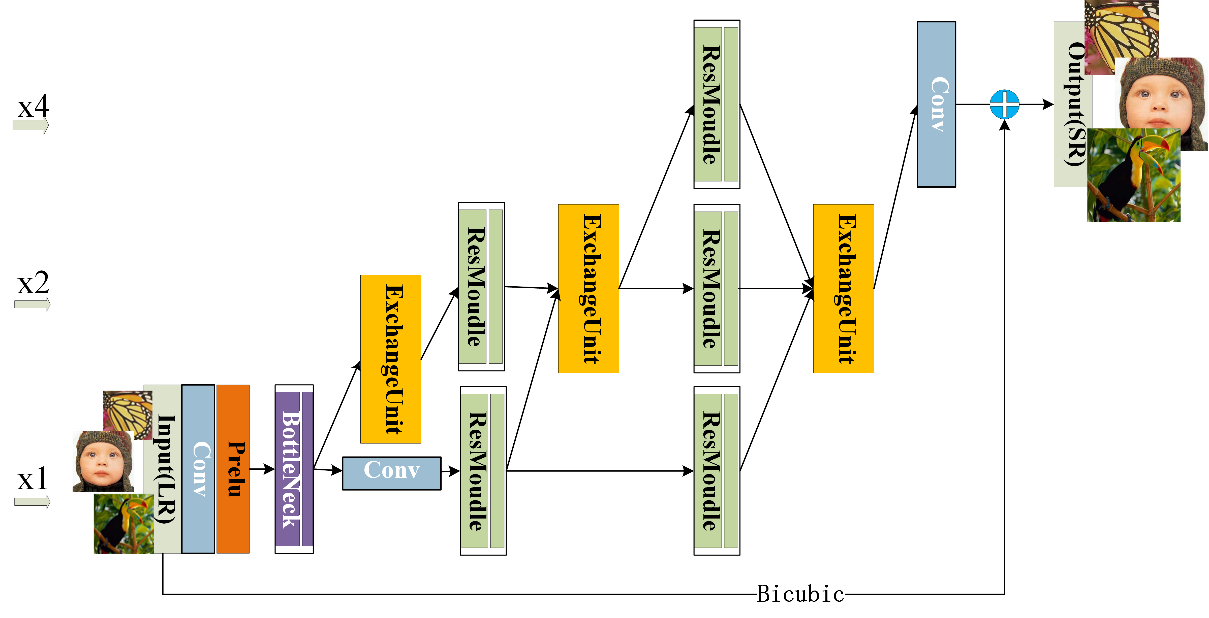


**Fig 1.** **The architecture of the multiresolution mixture network (MRMNet).** The x1, x2 and x4 levels denote the scale of the feature maps. The input dimension of the network is 24*24, with the subsequent output dimension being magnified by four to 96*96.

There are three types of resolution feature map (x1, x2, x4) in the whole network. The label x1 signifies that the resolution of the feature map is the same as the original resolution, whilst x2 and x4 denote that the resolution of the feature map is magnified two and four times the original resolution, respectively. The entire network gradually enlarges x1 resolution frames to x4 resolution in multiple stages.

Specifically, the MRMNet consists of three components: a bottleneck module, an exchange unit, and a residual module. The bottleneck module is responsible for feature extraction from LR frames and expressing LR features efficiently. The exchange unit is the central component of MRMNet, enlarging frames and obtaining exchanged features. The exchanged features have higher resolution feature maps that were obtained from other similar and lower resolution feature maps. These multiresolution feature maps are then combined to form a new feature map. The residual module is responsible for feature extraction and representation learning after the exchange unit.

The details of the bottleneck module are shown in Fig 2, consisting mainly of convolution layers and activation layers. The details of the convolution layers are also shown in Fig 2, with the kernel size (k), the number of channels (n), and stride (s) all given. In the activation layers, we chose the parametric rectified linear unit (PReLU) function as an activation function. The input and output of the bottleneck module are all LR feature maps. It should be noted that the feature map obtained by the input feature map through the single and triple convolution layer routes will be combined, then activated as the output of the bottleneck module.


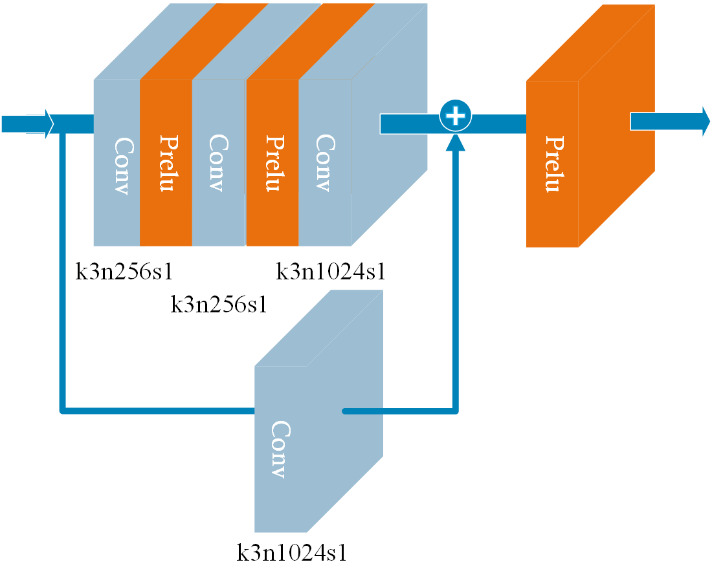


**Fig 2. The architecture of the bottleneck module of the MRMNet.** The kernel size (k), number of channels (n), and stride (s) of each convolutional layer are presented. The input dimension of the bottleneck module is [24, 24, *c*] and the output dimension is [24, 24, 1024], where *c* denotes the number of channels of the input feature maps.


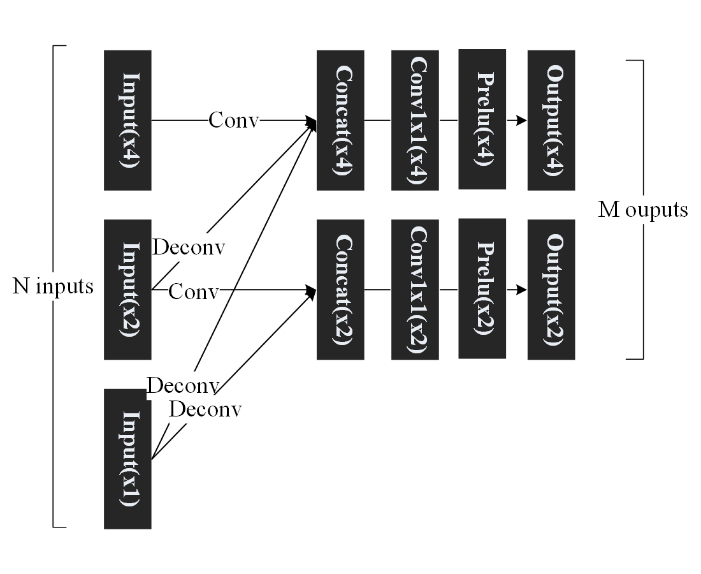


**Fig 3. The architecture of the exchange unit of the MRMNet.** The dimension of the input(x1), input(x2), and input(x4) are [24, 24, *c1*], [48, 48, *c2*], and [96, 96, *c4*] respectively. The dimension of the output(x2) and output(x4) are [48, 48, 64] and [96, 96, 32] respectively. The labels *c1*, *c2*, and *c4* denote the number of channels of corresponding feature maps.

The exchange unit of the MRMNet is shown in Fig 3. There are three exchange units in the MRMNet, with different numbers of feature maps as inputs and outputs. In Fig 3, we combine the three exchange units together, with *N* inputs and *M* outputs. The values of *N* and *M* are 3 and 2 respectively in Fig 3. The resolutions of the input feature maps are x1, x2, and x4, while the resolutions of the output feature maps are x2 and x4. The feature map of x1 resolution is enlarged to x2 resolution by the deconvolution operation and is enlarged to x4 resolution by applying the deconvolution operation twice. If the x1 resolution feature map was enlarged to x4 resolution through applying the deconvolution operation only once, the training results would produce checkerboard artifacts. Therefore, we use deconvolution twice in this scenario. The feature map of x2 resolution is enlarged to x4 resolution by applying the deconvolution operation once. In all deconvolution layers, the kernel size is 3x3, the stride is 2, and the number of channels of x1, x2, and x4 feature maps are 128, 64, and 32, respectively. We also can obtain the feature map with the same resolution as the input feature maps through the convolution operation, where kernel size is 3x3, the stride is 1, and the number of channels of x1, x2, and x4 feature maps are 128, 64, and 32, respectively. If someone needs to obtain a feature map with a specified resolution as output, they should receive all feature maps less than or the same as this resolution. For example, if the x4 resolution feature map is the desired output, the feature maps from x1 and x2 resolution feature maps must be obtained first, through deconvolution. Second, if the inputs of the exchange unit contain a x4 resolution feature map, a new feature map with the same resolution should be obtained from it via convolution. Then, all feature maps (if there are more than one) are merged into a feature map through the concatenation of feature maps in the channel dimension. The number of channels is adjusted through the convolution layer that has a kernel size of 1x1, a stride of 1, and the number of channels of x1, x2, and x4 feature maps are 128, 64, and 32, respectively. The final output is obtained using an activation layer. Through this structure, the exchange unit can supplement the information gathered from the LR frame and transfer it to the HR frame, obtain enlarged frames, and exchange features. After the first exchange unit, the number of channels of x1, x2, and x4 feature maps are 128, 64, and 32, respectively.

The residual module of MRMNet, as shown in Fig 4, is a classic residual network without the batch normalization (BN) layer. The MRMNet also has a convolutional layer and an activation layer at the beginning of network, and a convolutional layer after the bottleneck module and at the end of the network. At the beginning, the kernel size of the convolutional layer is 3x3, the number of channels is 64, and the stride is 1. After the bottleneck module, the kernel size is 3x3, the number of channels is 128, and the stride is 1. By the end, the size of the convolutional kernel is 9x9, the number of channels is 3, and the stride is 1.


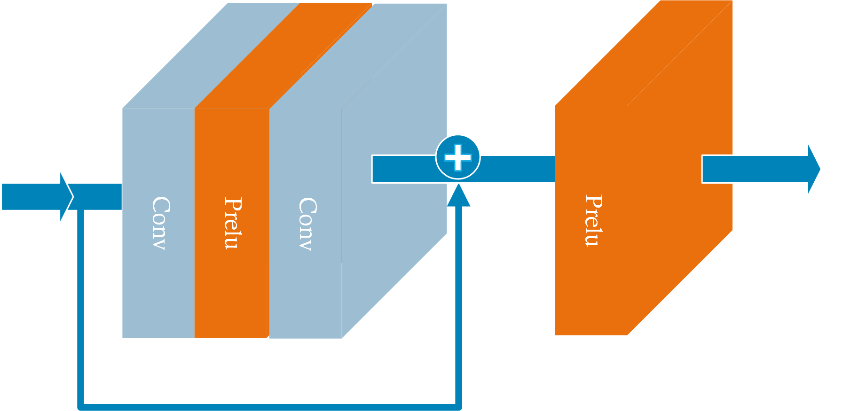


**Fig 4. The residual module of MRMNet.** The input dimension of the residual module is [*a, a, c*], and output is [*a, a, c*], where *a* denotes the length and width of the feature maps and *c* denotes the number of channels in the input feature maps. The parameters of the two convolution layers remain the same, where kernel size is 3x3 and the stride is 1. If the value of *a* is 24, 48, and 96, the number of channels are 128, 64, and 32, respectively.

In the MRMNet, the number of bottleneck modules and residual modules used in each stage can be adjusted. In this paper, the number of each component is fixed at two, in each stage.

**Loss function**

In deep learning-based methods, the loss function is necessary since it can guide the neural network to learn desired information. In order to reduce the overall fluctuation of residual values between the SR frame and HR frame, hence avoiding a result with excessive local differences, we propose a residual fluctuation loss function. We will introduce the details of the residual fluctuation loss and the final loss function as follows.

**Residual fluctuation loss function.** The residual fluctuation loss function $l_{rf}^{SR}$is proposed to reduce the overall fluctuation and avoid excessive local differences in the generated frame. Such a residual function mainly refers to the variance in probability theory and statistics. The function is calculated by determining the variance of the difference between the HR feature map and the SR feature map,

$l_{rf}^{SR}=\frac{1}{WH-1}\sum_{x=1}^{W} \sum_{y=1}^{H} {({Res}_{x,y}- mean(Res))}^{2}$ , (1)

where H and W denote the dimensions of the feature maps, $Res$ denotes the residual between the reference HR frame and the generated SR frame, and $mean(Res)$ denotes that the average value of pixels is calculated based on the obtained residual, $Res$. The residual, $Res$, can be calculated using,

${Res}_{VGGvgg}=\Phi_{VGGvgg}(I^{HR})-\Phi_{VGGvgg}(G_{\theta_{G}}\left( I^{LR} \right))$ . (2)

Here, $\Phi_{vgg}$ denotes the feature map that is obtained by the VGG-19 [[31](#_ENREF_31)] network,$I^{LR}$ is the LR frame version of its HR counterpart $I^{HR}$.

**Final loss function.** During training, the proposed model uses a residual fluctuation loss function in combination with the loss function of the generator $\mathcal{L}_{G,F-TecoGAN}$, as the final loss function of MRMVSR’s generator. The generator $\mathcal{L}_{G,F-TecoGAN}$ is calculated using,

$\mathcal{L}_{G,F-TecoGAN}=\sum\left\| g_{t}-y_{t} \right\|_{2}-\lambda_{a}\sum\log D_{s,t}\left( {IN}_{s,t}^{g} \right)$

$+\sum\lambda_{l}^{i}\left\| \Phi_{D_{s,t}}\left( {IN}_{s,t}^{g} \right)-\Phi_{D_{s,t}}\left( {IN}_{s,t}^{y} \right) \right\|_{2}$

$+\sum\lambda_{p}^{i}\left\| \Phi_{VGG}\left( g_{t} \right)-\Phi_{VGG}\left( y_{t} \right) \right\|_{2}$

$+ \lambda_{p}\mathcal{L}_{PP}+\lambda_{w}\mathcal{L}_{warp}$ . (3)

In (3), $g_{t}$and $y_{t}$ denote the generated frame and reference frame, respectively, while ${IN}_{s,t}^{g}$and ${IN}_{s,t}^{y}$ denote the generated frame and reference frame, respectively, as the inputs of discriminator. Furthermore, $\lambda$are the coefficients of the loss function, $\Phi_{D_{s,t}}$ and $\Phi_{VGG}$denote the discriminator network and VGG-19 network, respectively, and $\mathcal{L}_{PP}$ and $\mathcal{L}_{warp}$ are the Ping-Pong loss and *warp* loss that are consistent with TecoGAN [[11](#_ENREF_11)].

Using these two variables, the final loss function used for training is,

$\mathcal{L}_{G,F-MRMVSR}=\mathcal{L}_{G,F-TecoGAN}+\sum\lambda_{p}^{i}l_{rf}^{SR}$ （4）

# Results and discussion

**Datasets**

The training data used to test MRMVSR had the same source as the TecoGAN, which were obtained from the HR video dataset Vimeo [[32](#_ENREF_32)]. Specifically, this training set has 290 video clips that were extracted from 28 high-definition videos. Each video clip consists of 120 frames, and hence 34,800 images were included in the training set. We used 250 (a total of 30,000 images) and 40 (a total of 4800 images) video clips for training and validation processes, respectively. Image augmentation was used in the training process, including rotations and flipping.

Four scene sets in the Vid4 dataset [[26](#_ENREF_26)] were used as the test data in both the MRMVSR model and the comparative models; namely Calendar, City, Foliage, and Walk. These four scene data sets all contain 50 consecutive video frames, which are commonly used in the field of VSR.

**Training details**

In the training process, following the TecoGAN model, the MRMVSR model amplifies the video frame resolution four times to obtain the SR video frames. The LR video frame was obtained by downsampling the HR video frame, implemented via bicubic interpolation. The LR video frame was normalized to [0, 1], whilst the HR video frame was normalized to [-1, 1]. To prevent the generation of local noise, the feature map was cropped to [-5, 5] after the exchange unit.

The training process had two stages, with each stage comprising 500,000 steps. Each batch contained four different videos. Ten consecutive frames were selected for each video, so each batch consisted of 40 frames. The LR images were cropped to the same size of 24x24. The first stage was a pre-training stage without discriminator, where the loss function was $\sum\left\| g_{t}-y_{t} \right\|_{2}$+ $\lambda_{w}\mathcal{L}_{warp}$. The second stage was a formal training stage, which used the generative adversarial network with a discriminator. The learning rate in both stages was set as 5e-5. Adam was chosen as the optimization algorithm, where $\beta_{1}$ and $\beta_{2}$ were 0.9 and 0.99, respectively. The final loss function used was $\mathcal{L}_{G,F-MRMVSR}$, where $\lambda_{a}$= 0.01, $\lambda_{p}=\lambda_{w}$= 1, $\lambda_{l}^{i}$= 0.02, $\lambda_{p}^{i}$= 1.

The model was implemented in Python3.5 and Tensorflow1.10. Matplotlib3.0.3, Numpy1.14.5, and Opencv-python 4.1.0.25 were also used. The memory of the GPU used was 16GB.

**Experimental results**

To demonstrate the performance of the MRMVSR method, we made several experimental comparisons analyzing three main aspects: the network, loss function, and overall performance of the model. The details are described separately as follows.

**Network performance.** To verify the performance of the MRMNet, the generator of TecoGAN-G was selected as the comparative network. The loss functions $\mathcal{L}_{G,F-TecoGAN}$ of TecoGAN and $\mathcal{L}_{G,F-MRMVSR}$ of MRMNet were used to train the generator. Learned perpetual image patch similarity (LPIPS) [[33](#_ENREF_33)] and (tLP) [[11](#_ENREF_11)] were selected as the evaluation metrics on the Vid4 test set, for measuring spatial detail and temporal coherence respectively. A smaller value represents an improved performance.

We took $\mathcal{L}_{G,F-TecoGAN}$ as the generator loss function to evaluate the network performance at first. Fig 5 and Fig 6 show the evaluation results according to the LPIPS metric and tLP metric, respectively.


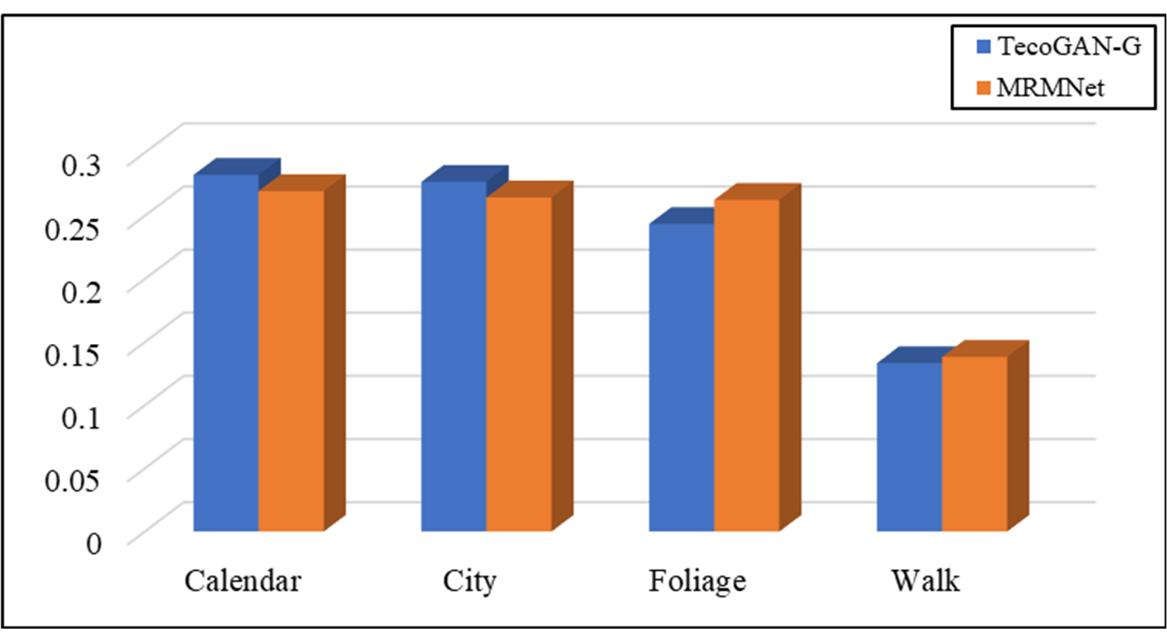


**Fig 5. Evaluation results using the LPIPS metric for MRMNet and TecoGAN-G on four different data sets.** The loss function used is $\mathcal{L}_{G,F-TecoGAN}$.


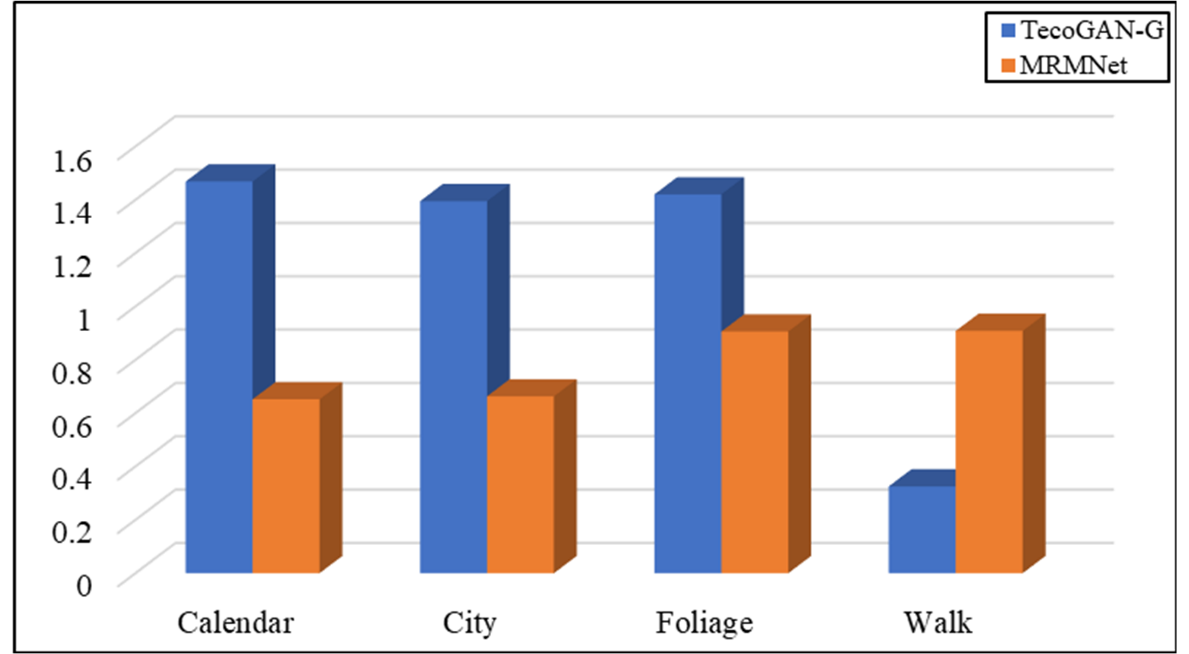


**Fig 6. Evaluation results using the tLP metric for MRMNet and TecoGAN-G on four different data sets.** The loss function used is $\mathcal{L}_{G,F-TecoGAN}$.

From Fig 5, we can observe that TecoGAN-G and MRMNet have their own advantages on the four different scene datasets when using the same loss function according to the LPIPS metric. The evaluation results indicate that both networks can generate SR video with better visual quality. From Fig 6, we can observe that the performance of MRMNet is better than TecoGAN-G according to the tLP metric, significantly outperforming the other method in three out of the four data sets. This indicates that the SR video derived from MRMNet has an improved visual perception quality whilst also having better continuity. From the above conclusions, the performance of MRMNet can be considered to be better than TecoGAN-G.

In addition, we define $\mathcal{L}_{G,F-MRMVSR}$ as the loss function of the generator and conduct contrast experiments to evaluate the network performance. Fig 7 shows the evaluation results according to the LPIPS metric, while Fig 8 shows the evaluation results according to the tLP metric.


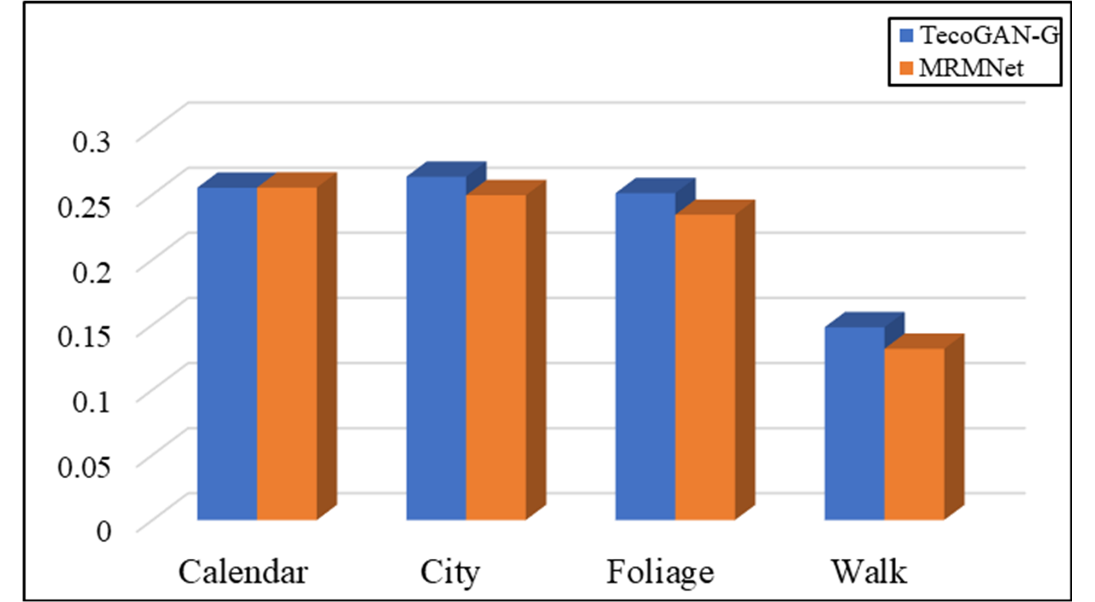


**Fig 7. The evaluation results of MRMNet and TecoGAN-G on four different data sets, using the LPIPS metric.** $\mathcal{L}_{G,F-MRMVSR}$ is the loss function used.


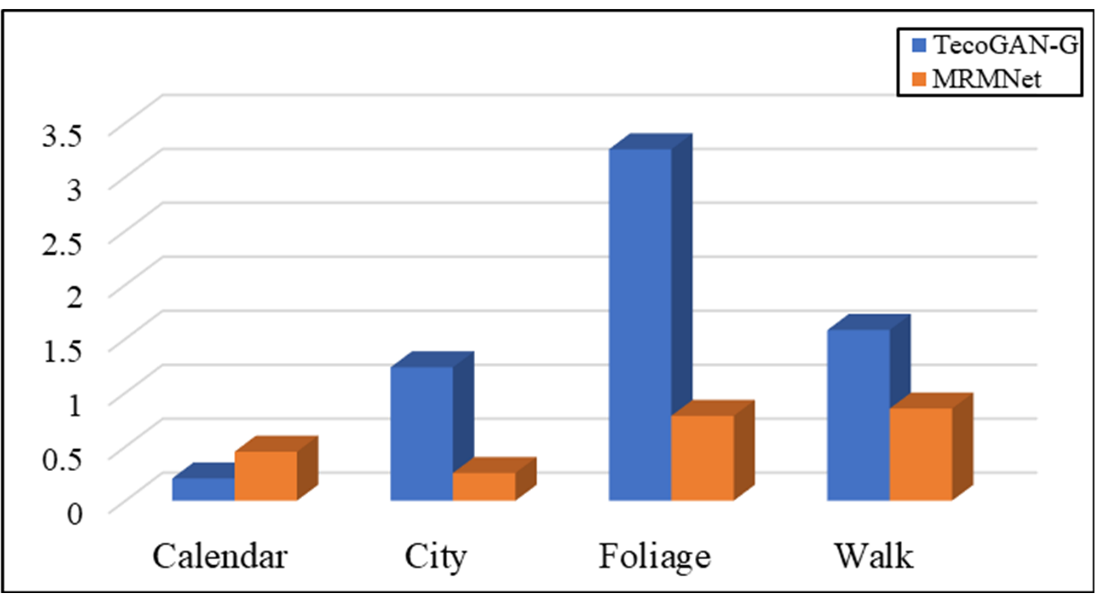


**Fig 8. The evaluation results of MRMNet and TecoGAN-G on four different data sets, using the tLP metric.** $\mathcal{L}_{G,F-MRMVSR}$ is the loss function used.

As shown in Fig 7, according to the LPIPS metric, the MRMNet with loss function $\mathcal{L}_{G,F-MRMVSR}$ is better than TecoGAN-G with the same loss function in different test sets. This means that MRMNet can produce videos with a higher visual quality than the other method. Fig 8 shows that the performance of MRMNet is also better than that of TecoGAN-G network according to the tLP metric. It can be found that the SR video generated by MRMNet has better visual perception quality and continuity than the video generated by TecoGAN-G. In summary, the MRMNet performs better compared to TecoGAN-G.

According to the above experiments, it is shown that our MRMNet is superior to TecoGAN-G in both visual perception quality and continuity.

**Performance results and analysis of loss function.** In order to show the effectiveness of the proposed residual fluctuation loss function, we use MRMNet as the generator network with different loss functions, $\mathcal{L}_{G,F-TecoGAN}$ and $\mathcal{L}_{G,F-MRMVSR}$. Vid4 was used again as the evaluation dataset, while LPIPS and tLP were again selected as evaluation metrics. Figs 9 and 10 show the experimental evaluation results, using the LPIPS metric and tLP metric respectively to compare the effectiveness of the loss functions.


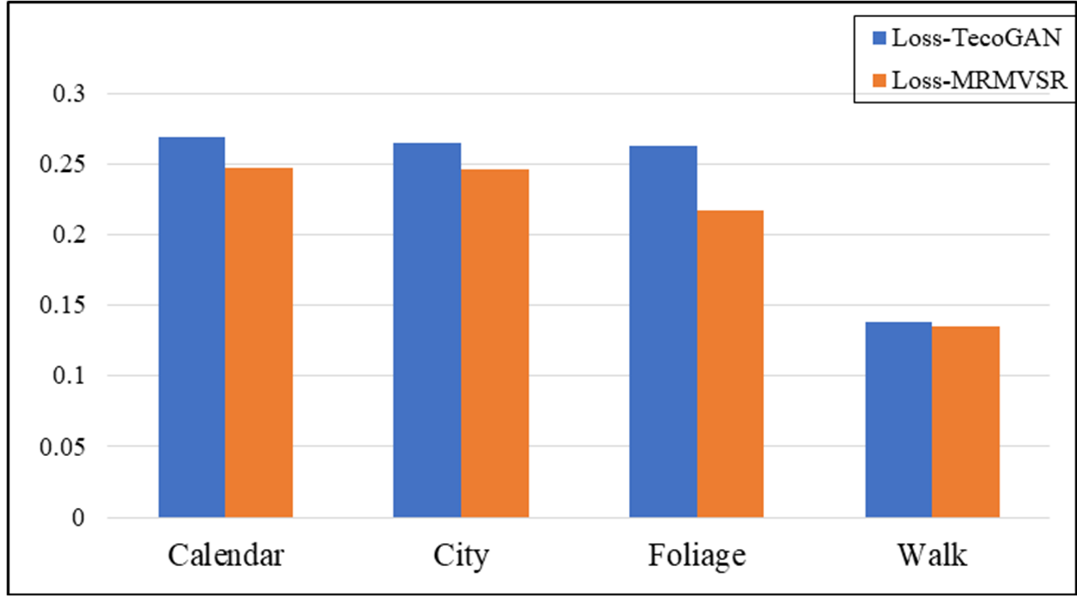


**Fig 9. The evaluation results comparing different loss functions on four different data sets, according to the LPIPS metric.** Loss-TecoGAN represents the loss function $\mathcal{L}_{G,F-TecoGAN}$, whilst Loss-MRMVSR represents the loss function $\mathcal{L}_{G,F-MRMVSR}$.


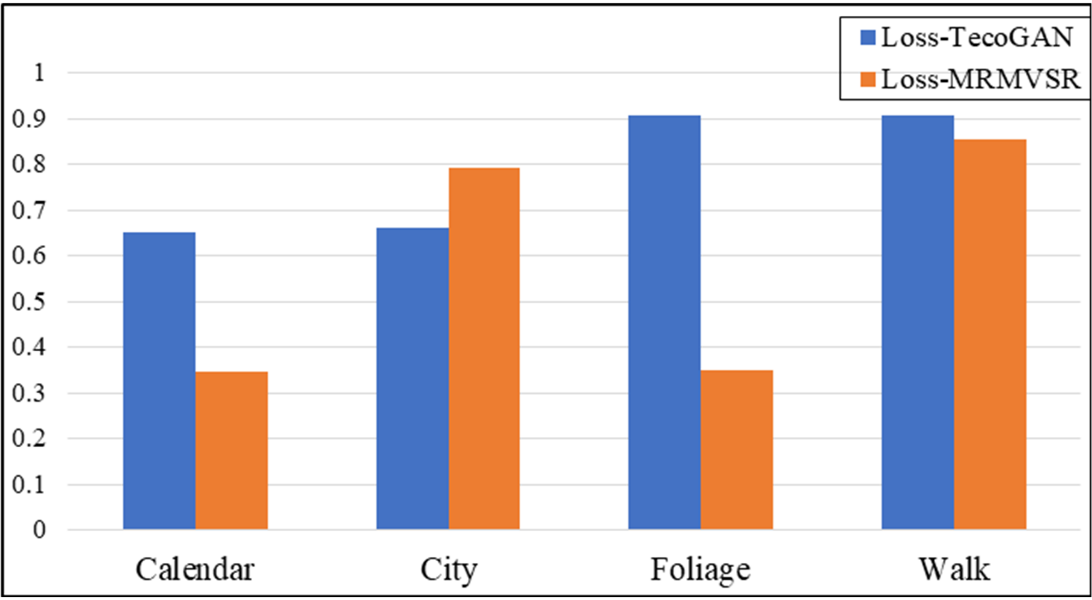


**Fig 10. The evaluation results comparing different loss functions on four different data sets.** Loss-TecoGAN represents the loss function $\mathcal{L}_{G,F-TecoGAN}$, whilst Loss-MRMVSR represents the loss function $\mathcal{L}_{G,F-MRMVSR}$.

We can observe that the result generated by the proposed residual fluctuation loss function $\mathcal{L}_{G,F-MRMVSR}$ has a smaller LPIPS value compared to different loss functions from Fig 9. This shows that adding a residual fluctuation loss function can effectively improve the visual perception quality of SR video. From the results of Fig 10, the model with our proposed loss function $\mathcal{L}_{G,F-MRMVSR}$ can also generate a smaller tLP value in a majority of data sets. This indicates that the generated video from the proposed method has better continuity features. In summary, adding a residual fluctuation loss function can effectively improve the performance of VSR, with both the visual perception quality and video continuity improved.

**Results and analysis of overall performance of the model.** In Table 1, the evaluation results of MRMVSR compared to Bicubic, dynamic upsampling filter (DUF)[[34](#_ENREF_34)], FRVSR[[29](#_ENREF_29)], and TecoGAN[[11](#_ENREF_11)] methods are given, according to the LPIPS and tLP metrics.

**Table 1. Comparison of evaluation results of Bicubic, DUF, FRVSR, TecoGAN and MRMVSR methods.** The evaluation metrics are LPIPS and tLP. The lowest values are highlighted in bold, representing the best performances for each data set and evaluation metric permutation.

| Data set | Metric | Bicubic | DUF[[34](#_ENREF_34)] | FRVSR[[29](#_ENREF_29)] | TecoGAN[[11](#_ENREF_11)] | MRMVSR (proposed method) |
| --- | --- | --- | --- | --- | --- | --- |
| Calendar | LPIPS  tLP | 0.5676 | 0.3882 | 0.3027 | 0.2825 | **0.2473** |
|  |  | 3.1539 | 1.9293 | 1.1050 | 1.4686 | **0.3472** |
| City | LPIPS  tLP | 0.5208 | 0.3499 | 0.3532 | 0.2769 | **0.2467** |
|  |  | 2.3497 | 1.9936 | 1.9411 | 1.3946 | **0.7923** |
| Foliage | LPIPS  tLP | 0.5459 | 0.4109 | 0.4124 | 0.2436 | **0.2177** |
|  |  | 4.4131 | 2.1364 | 2.4091 | 1.4203 | **0.3512** |
| Walk | LPIPS  tLP | 0.3694 | 0.1897 | 0.1992 | **0.1334** | 0.1353 |
|  |  | 1.3073 | 0.2132 | **0.1621** | 0.3254 | 0.8537 |

As shown in Table 1, the proposed method MRMVSR achieved the best performance in most scenarios according to the tLP metric and LPIPS metric, scoring the lowest evaluation results for three of the four data sets. This implies that the proposed MRMVSR method can produce videos with better visual quality, as well as better continuity between video frames compared to the other tested methods.

In order to further demonstrate the performance of the MRMVSR model, we select one frame from the four different scenes: Calendar, City, Foliage, and Walk. The qualitative results are shown in Fig 11. Moreover, we enlarge a random part of the frames and compare them with other methods in terms of the LPIPS metric. From these images, we can observe that the proposed method obtained better results in the texture-intensive areas compared to alternative state-of-the-art methods.

# Conclusion

In this paper, we proposed an MRMVSR method with a new generative network and a residual fluctuation loss function for VSR tasks, with the proposed generative network called MRMNet. In this network, the HR feature map can continuously extract LR feature map information to supplement the images. The LR feature map is gradually enlarged to obtain the target resolution, hence the utilization rate of the LR feature map information is improved. The proposed residual fluctuation loss function is able to restrict large variations in the quality of the generated images, to avoid the large local differences. By comparing the MRMVSR method with other state-of-the-art models using the LPIPS and tLP metrics over four test data sets, the experimental results showed that the proposed method offers a significant improvement. The proposed method has the ability to generate videos with better visual perception quality and temporal coherence, significantly improving performance in areas with dense textures.


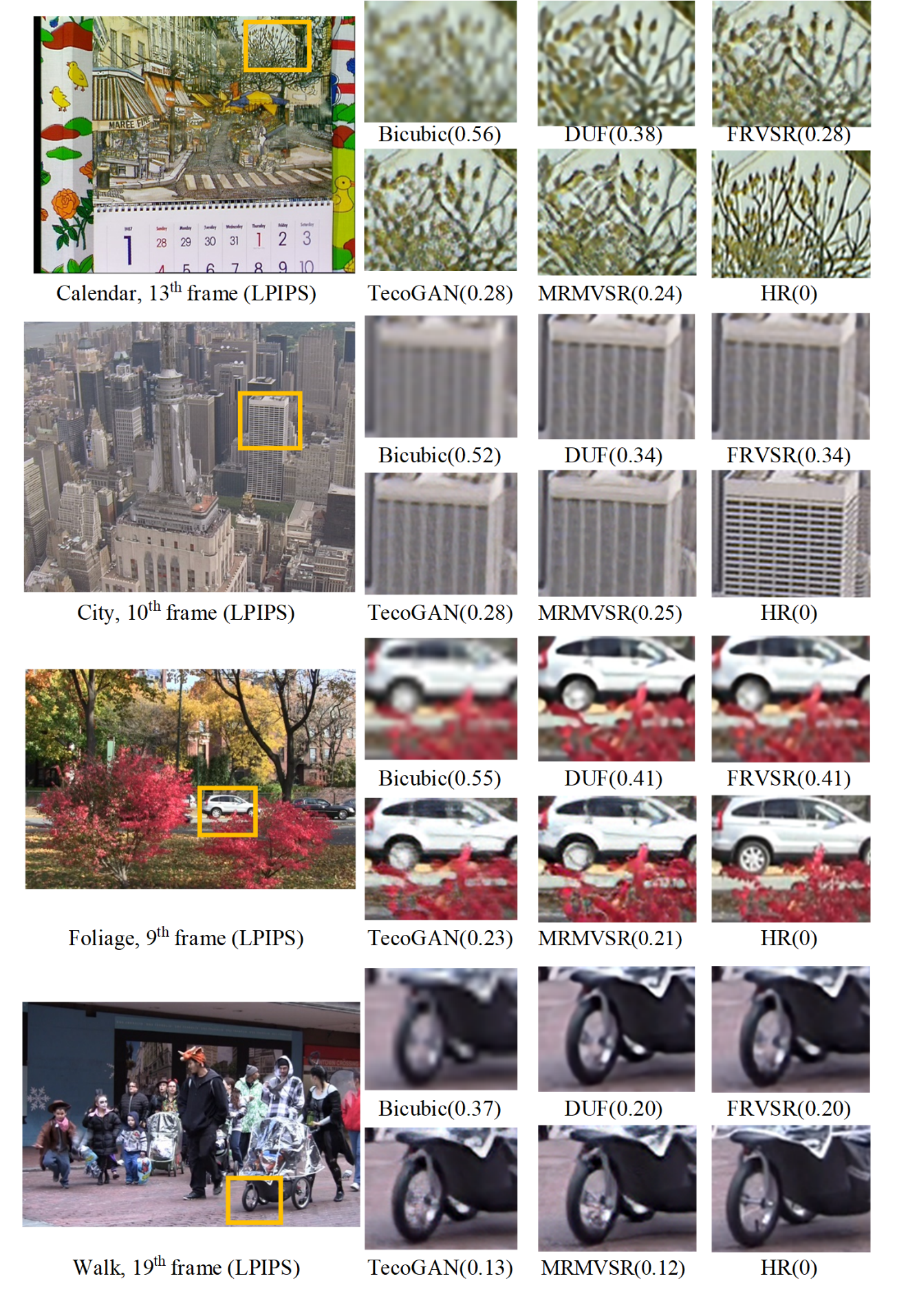


**Fig 11. Visual quality results of the MRMVSR method from the four test sets, compared to other state-of-the-art methods.**

# Acknowledgements

This work was supported in part by the key projects of Shaanxi province No. 2018ZDCXLGY0607, the Fundamental Research Funds for the Central Universities No. XJJ2018254, the China Postdoctoral Science Foundation No. 2018M631164, and the program of the Ministry of Education for the university.

# Author Contributions

Conceptualization: Zhiqiang Tian, Yudiao Wang, Shaoyi Du, Xuguang Lan.

Data curation: Yudiao Wang.

Formal analysis: Zhiqiang Tian, Yudiao Wang, Shaoyi Du.

Funding acquisition: Zhiqiang Tian, Xuguang Lan.

Investigation: Zhiqiang Tian.

Methodology: Zhiqiang Tian, Yudiao Wang, Shaoyi Du, Xuguang Lan.

Software: Yudiao Wang.

Supervision: Xuguang Lan.

Validation: Zhiqiang Tian, Yudiao Wang.

Visualization: Yudiao Wang, Shaoyi Du.

Writing – original draft: Zhiqiang Tian.

Writing – review & editing: Zhiqiang Tian, Yudiao Wang.

# References

1. Caballero J, Ledig C, Aitken AP, Acosta A, Totz J, Wang Z, et al. Real-Time Video Super-Resolution with Spatio-Temporal Networks and Motion Compensation. Proceedings of the IEEE conference on computer vision and pattern recognition; 2017. p. 4778-4787.

2. Dong C, Loy CC, He K, Tang X. Learning a deep convolutional network for image super-resolution. European conference on computer vision: Springer; 2014. p. 184-199.

3. Kappeler A, Yoo S, Dai Q, Katsaggelos AK. Video super-resolution with convolutional neural networks. IEEE Transactions on Computational Imaging. 2016;2(2):109-122.

4. Kim J, Kwon Lee J, Mu Lee K. Accurate image super-resolution using very deep convolutional networks. Proceedings of the IEEE conference on computer vision and pattern recognition; 2016. p. 1646-1654.

5. Ledig C, Theis L, Huszár F, Caballero J, Cunningham A, Acosta A, et al. Photo-realistic single image super-resolution using a generative adversarial network. Proceedings of the IEEE conference on computer vision and pattern recognition; 2017. p. 4681-4690.

6. Sajjadi MS, Scholkopf B, Hirsch M. Enhancenet: Single image super-resolution through automated texture synthesis. Proceedings of the IEEE International Conference on Computer Vision; 2017. p. 4491-4500.

7. Liu D, Wang Z, Fan Y, Liu X, Wang Z, Chang S, et al. Robust Video Super-Resolution with Learned Temporal Dynamics. Proceedings of the IEEE conference on international conference on computer vision; 2017. p. 2526-2534.

8. Shi W, Caballero J, Huszár F, Totz J, Aitken AP, Bishop R, et al. Real-time single image and video super-resolution using an efficient sub-pixel convolutional neural network. Proceedings of the IEEE conference on computer vision and pattern recognition; 2016. p. 1874-1883.

9. Liu Z. Cross-View Multi-Lateral Filter for Compressed Multi-View Depth Video. IEEE Transactions on Image Processing. 2019;28(1):302-315.

10. Goodfellow I, Pouget-Abadie J, Mirza M, Xu B, Warde-Farley D, Ozair S, et al. Generative adversarial nets. Advances in neural information processing systems; 2014. p. 2672-2680.

11. Chu M, Xie Y, Leal-Taixé L, Thuerey N. Temporally Coherent GANs for Video Super-Resolution (TecoGAN). ACM SIGGRAPH. 2020:1-20.

12. Yang Y, Li B, Li P, Liu Q. A Two-Stage Clustering Based 3D Visual Saliency Model for Dynamic Scenarios. IEEE Transactions on Multimedia. 2019;21(4):809-820.

13. An P, Liu Q, Abedi F, Yang Y. Novel calibration method for camera array in spherical arrangement. Signal Processing-image Communication. 2020;80:115682.

14. Abedi F, Yang Y, Liu Q. Group geometric calibration and rectification for circular multi-camera imaging system. Optics Express. 2018;26(23):30596-30613.

15. Dong C, Loy CC, He K, Tang X. Image super-resolution using deep convolutional networks. IEEE transactions on pattern analysis and machine intelligence. 2015;38(2):295-307.

16. Wang Z, Chen J, Hoi SC. Deep learning for image super-resolution: A survey. IEEE Transactions on Pattern Analysis and Machine Intelligence. 2020:1-24.

17. Dong C, Loy CC, Tang X. Accelerating the super-resolution convolutional neural network. European conference on computer vision: Springer; 2016. p. 391-407.

18. Ghifary M, Kleijn WB, Zhang M, Balduzzi D, Li W. Deep reconstruction-classification networks for unsupervised domain adaptation. European Conference on Computer Vision: Springer; 2016. p. 597-613.

19. Lim B, Son S, Kim H, Nah S, Mu Lee K. Enhanced deep residual networks for single image super-resolution. Proceedings of the IEEE conference on computer vision and pattern recognition workshops; 2017. p. 136-144.

20. Haris M, Shakhnarovich G, Ukita N. Deep back-projection networks for super-resolution. Proceedings of the IEEE conference on computer vision and pattern recognition; 2018. p. 1664-1673.

21. Johnson J, Alahi A, Fei-Fei L. Perceptual losses for real-time style transfer and super-resolution. European conference on computer vision: Springer; 2016. p. 694-711.

22. Wang X, Yu K, Wu S, Gu J, Liu Y, Dong C, et al. ESRGAN: Enhanced Super-Resolution Generative Adversarial Networks. European conference on computer vision; 2018. p. 63-79.

23. Wang X, Yu K, Dong C, Change Loy C. Recovering realistic texture in image super-resolution by deep spatial feature transform. Proceedings of the IEEE Conference on Computer Vision and Pattern Recognition; 2018. p. 606-615.

24. Zhang Z, Wang Z, Lin Z, Qi H. Image Super-Resolution by Neural Texture Transfer. Proceedings of the IEEE Conference on Computer Vision and Pattern Recognition; 2019. p. 7982-7991.

25. Pathak HN, Li X, Minaee S, Cowan B. Efficient Super Resolution for Large-Scale Images Using Attentional GAN. 2018 IEEE International Conference on Big Data (Big Data): IEEE; 2018. p. 1777-1786.

26. Liu C, Sun D. A bayesian approach to adaptive video super resolution. Proceedings of the IEEE conference on computer vision and pattern recognition: IEEE; 2011. p. 209-216.

27. Takeda H, Milanfar P, Protter M, Elad M. Super-Resolution Without Explicit Subpixel Motion Estimation. IEEE Transactions on Image Processing. 2009;18(9):1958-1975.

28. Makansi O, Ilg E, Brox T. End-to-End Learning of Video Super-Resolution with Motion Compensation. German conference on pattern recognition; 2017. p. 203-214.

29. Sajjadi MS, Vemulapalli R, Brown M. Frame-recurrent video super-resolution. Proceedings of the IEEE Conference on Computer Vision and Pattern Recognition; 2018. p. 6626-6634.

30. You X, Franz E, Chu M, Thuerey N. tempoGAN: A Temporally Coherent, Volumetric GAN for Super-resolution Fluid Flow. ACM Transactions on Graphics. 2018;37(4):1-15.

31. Szegedy C, Vanhoucke V, Ioffe S, Shlens J, Wojna Z. Rethinking the Inception Architecture for Computer Vision. Proceedings of the IEEE conference on computer vision and pattern recognition; 2016. p. 2818-2826.

32. Wang X, Chan KCK, Yu K, Dong C, Loy CC. EDVR: Video Restoration With Enhanced Deformable Convolutional Networks. Proceedings of the IEEE conference on computer vision and pattern recognition; 2019. p. 1-10.

33. Zhang R, Isola P, Efros AA, Shechtman E, Wang O. The unreasonable effectiveness of deep features as a perceptual metric. Proceedings of the IEEE Conference on Computer Vision and Pattern Recognition; 2018. p. 586-595.

34. Jo Y, Wug Oh S, Kang J, Joo Kim S. Deep video super-resolution network using dynamic upsampling filters without explicit motion compensation. Proceedings of the IEEE conference on computer vision and pattern recognition; 2018. p. 3224-3232.
