# Supplementary material for: A multiresolution mixture generative adversarial network for video super-resolution
Source: PLoS One. 2020 Jul 10;15(7):e0235352. doi: 10.1371/journal.pone.0235352 (PMC7351143; doi:10.1371/journal.pone.0235352)
Supplement: S2 Data — (ZIP) [file pone.0235352.s002.zip › Editage Editing Service/Title_Page_-_ONE_Formatting.pdf]

TITLE, AUTHOR, AFFILIATIONS FORMATTING GUIDELINES

Symbol Legend		
**Symbol**	**Name**	**Definition**
¶	Pilcrow (paragraph symbol)	1st set of equal contributors
&	Ampersand	2nd set of equal contributors
*	Asterisk	Corresponding author(s)
#a	Pound/number sign	First Current address
#b	Pound/number sign	Second Current address
†	Dagger/Cross	Deceased
^	Caret	Consortium/Group Authorship

This is the article title

John Doe[1¶], Antonie Data[1¶], Johannes van Stats[1,#a], Marie Testperson[2*], David Ribosome Jr.[3,4], Gregory H.T. McBio[5,#b], Angela Reviewerson[1,2&], Marina Measure[1&], on behalf of The Bunny Genome Sequencing Consortium[^]

[1] Department, Institution, City, State, Country

[2] Department of Dermatology, Division of Rabbit Health, Section of Veterinary Medicine, St. Hare Hospital, San Francisco, California, United States of America

[3] Department of Libraries and Archives, National Contemporary Bunny Museum, Lagomorph, Connecticut, United States of America

[4] Department of Restoration, National Contemporary Bunny Museum, Lagomorph, Connecticut, United States of America

[5] Department of Archaeology, Bunny University, Lagomorph, Connecticut, United States of America

[#a]Current Address: Department of Carrot Science, Bunny University, Lagomorph, Connecticut, United States of America

[#b]Current Address: Department of Canine Evasion, Bunny University, Lagomorph, Connecticut, United States of America

* Corresponding author

E-mail: testperson@university.ed (MT)

[¶]These authors contributed equally to this work.

[&]These authors also contributed equally to this work.

[^]Membership of the Bunny Genome Sequencing Consortium is provided in the Acknowledgments.

Article Title

- Italics, bold type, symbols, and other text formatting will all be reproduced in the published article as submitted.
- Titles should be written in sentence case (capitalize only the first word of the title, the first word of the subtitle, and any proper nouns and genus names).

Author Byline

- Author names will be published exactly as they appear in the accepted manuscript.
- Indicate affiliations by number only.
- Affiliation footnotes should appear in numerical order at first mention.
- Please use the symbols provided in this document for other designations.
- Numbers and symbols should be in superscript.
- Do not include titles (Dr., PhD, Professor, etc.).

Affiliations

- Affiliations will be published as they appear in the accepted manuscript.
- Include each component in order of small to large (Department, Division, Section, Institution, City, State, Country).
- Do not include ZIP or Postal Codes, street addresses, or building/office numbers.
- Do not use abbreviations (e.g. Dept.).
- Do not list positions within an institution (e.g. Department Chair, Professor, etc.).
- List each affiliation individually and in full.

Corresponding Authorship

- Do not include physical addresses; only email addresses are required.
- List corresponding author's initials in parentheses after the email address.

Contributorship

- Use the symbols provided here to indicate equal contributions.
- If you would like the equal contributions notes to read differently, please specify in your manuscript (e.g., "AR and MM are Joint Senior Authors").

Consortia or other Group Authors

- If there is a consortium or group author on your manuscript, please provide a note that describes where the full membership list is available for the readers.
- The membership list can be listed in the Acknowledgments, in Supporting Information, or on the internet.
- Consortia/Group authors can have affiliations, but it is not required.
